# Effects of virtual reality associated with serious games for upper limb rehabilitation inpatients with multiple sclerosis: randomized controlled trial

**DOI:** 10.1186/s12984-020-00718-x

**Published:** 2020-07-13

**Authors:** Alicia Cuesta-Gómez, Patricia Sánchez-Herrera-Baeza, Edwin Daniel Oña-Simbaña, Alicia Martínez-Medina, Carmen Ortiz-Comino, Carlos Balaguer-Bernaldo-de-Quirós, Alberto Jardón-Huete, Roberto Cano-de-la-Cuerda

**Affiliations:** 1 Department of Physical Therapy, Occupational Therapy, Physical Medicine and Rehabilitation, Faculty of Health Sciences, Rey Juan Carlos University, Avenida de Atenas s/n 28922 Alcorcón, Madrid, Spain; 2 Robotics Lab. University Carlos III of Madrid, Leganés, Madrid, Spain; 3 Asociación de Esclerosis Múltiple de Toledo (ADEMTO), Toledo, Spain

**Keywords:** Dexterity, Leap motion controller, Multiple sclerosis, Rehabilitation, Upper limb, Serious games, Virtual reality

## Abstract

**Background:**

Dexterity and activities of daily living limitations on the upper limb (UL) represent one of the most common problems in patients with multiple sclerosis (MS). The aim of this study was to evaluate the effectiveness of the specially developed Serious Games that make use of the Leap Motion Controller (LMC) as main user interface for improving UL grip muscle strength, dexterity, fatigue, quality of life, satisfaction and compliance.

**Methods:**

A single-blinded randomized controlled trial was conducted. The sample was randomized into two groups: an experimental group who received treatment based on serious games designed by the research team using the developed LMC based Serious Games for the UL plus conventional rehabilitation, and a control group who received the same conventional rehabilitation for the UL. Both groups received two 60 min sessions per week over a ten-week period. Grip muscle strength, coordination, speed of movements, fine and gross UL dexterity, fatigue, quality of life, satisfaction and compliance were assessed in both groups pre-treatment, post-treatment and in a follow-up period of 1 month without receiving any treatment.

**Results:**

In the experimental group compared to the control group, significant improvements were observed in the post-treatment assessment for coordination, speed of movements, fine and gross UL dexterity. Also, significant results were found in the follow-up in coordination, speed of movements, fine and gross for the more affected side.

**Conclusions:**

An experimental protocol using an LMC based Serious Games designed for UL rehabilitation showed improvements for unilateral gross manual dexterity, fine manual dexterity, and coordination in MS patients with high satisfaction and excellent compliance.

**Trial registration:**

This randomized controlled trial has been registered at ClinicalTrials.gov Identifier: NCT04171908, Nov 2019.

## Introduction

Multiple sclerosis (MS) is a chronic inflammatory demyelinating illness of the central nervous system of unknown etiology, currently representing the most common neurological illness causing disability among young adults in Europe and North America [1]. Common symptoms include fatigue, visual disorders, problems affecting balance and coordination, sensitivity disorders, spasticity, cognitive and emotional disorders, speech disorders, problems affecting the bladder and intestines, and sexual-related dysfunction [2].

Dexterity and activities of daily living (ADL) limitations on the upper limb (UL) represent one of the most common problems in patients with MS (4). After 15 years of disease evolution, the majority of those affected by MS report problems at the functional level in the hand, and patients are forced to make compensations or decrease activity in the functions that include the UL [3]. In addition, many studies link the decrease in independence in ADL with the loss of dexterity and manual coordination [3–5].

Despite the importance of UL performance in daily functional activities, it usually remains in the background in the rehabilitation of individuals with MS, giving more prominence to the rehabilitation of the lower limbs and balance [3]. Furthermore, rehabilitation treatments for patients with MS are described as very lengthy and systematic, leading to loss of motivation and compliance [6].

As a result, in recent years, technology-based rehabilitation systems, such as virtual reality (VR), are promising and may be able to deliver a client-centered task-oriented rehabilitation without requiring a device or controller [7]. Several studies have addressed the positive effects of VR systems as being a complementary therapy to neurological rehabilitation [6, 8]. These novel approaches enhance patient motivation by enabling the practice of functional tasks in virtual surroundings, providing patient feedback concerning results, all of which is based on the repetition of ADLs, facilitating motor learning and neuroplasticity through increased intensity during task-oriented training [6]. Thus, rehabilitation professionals have expanded the care of patients with MS, by including this technology as a complement to rehabilitation programs, achieving a higher treatment intensity at a sustainable cost [6]. However, few studies exist on the effects that VR has on the manual dexterity of patients with MS [6, 9].

Video games based on VR technology (i.e. Nintendo Wii, PlayStation Move, and Kinect plus XBOX 360) are emerging as valid tools used in neurorehabilitation for patients with MS. However, often these are either too difficult for patients or the games progress too quickly, failing to provide impairment-focused training or to specifically address patient needs [7]. Therefore, it is necessary to develop specific serious games for MS patients. Serious games are defined as games designed for a primary purpose other than that of pure entertainment, and which promote learning and behavior changes in MS patients. In this context, new low-cost markerless devices have emerged, such as the Leap Motion Controller (LMC) System®, which uses a sensor that captures the movement of the patient’s forearms and hands without the need to place sensors or devices on the body. This generates a virtual image of the UL on a computer screen, and the patient is prompted to perform movements according to the functional task proposed. This system presents important advantages over other motion capture systems, namely thanks to its portability, ease of use, commercial availability, low cost, and non-invasive nature [7]. However, evidence is lacking to support the therapeutic use of LMC in the treatment of UL motor disorders in MS. Furthermore, to our knowledge, no specific serious games have been designed for MS patients using the LMC system.

## Aim

The primary aim of the present study was to evaluate the effectiveness of LMC based Serious Games designed for neurological diseases for improving UL grip muscle strength, coordination, speed of movements, fine and gross dexterity, fatigue, and quality of life. Furthermore, we sought to assess satisfaction and compliance levels in MS patients.

## Methods

### Design

A single-blinded randomized controlled trial (RCT) was conducted (NCT04171908) following the CONsolidated Standards of Reporting Trials (CONSORT) guidelines. Non-probabilistic sampling of consecutive cases was used. The sample was randomized, after using QuickCalcs GraphPad Software, into a control group (CG) who received conventional rehabilitation treatment and an experimental group (EG) who received VR treatment with LMC in addition to their conventional rehabilitation sessions. All interventions were performed at the Leganés Association of Multiple Sclerosis and Toledo Association of Multiple Sclerosis in Madrid and Toledo (Spain).

### Participants

The study inclusion criteria were as follows: a diagnosis of MS according to the McDonald criteria [2] with over 2 years evolution; a score of between 3.5 (moderate incapacity, although totally ambulant, self-sufficient, and active for 12 h/day) and 7.5 (unable to take more than a few steps and may need aid in transferring. Can wheel self but cannot carry on in standard wheelchair for a full day and may require a motorized wheelchair) on the Kurtzke Expanded Disability Status Scale (EDSS); with stable medical treatment during at least the 6 months prior to the intervention; muscle tone in the upper limbs not greater than two points on the modified Ashworth Scale (moderate hypertonia, increased muscle tone through most of the range of movement, but affected part easily moved); as well as a score of four points or less in the “Pyramidal Function” section of the EDSS functional scale; absence of cognitive decline; with the ability to understand instructions and obtaining a score of 24 or more in the Mini-Mental Test; and a score of two points or less in the “Mental Functions” section of the EDSS.

The exclusion criteria were a diagnosis of another neurological illness or musculoskeletal disorder different to MS; the diagnosis of a cardiovascular, respiratory, or metabolic illness or other conditions which may interfere with the study; suffering a flare-up or hospitalization in the last 3 months prior to commencement of the assessment protocol or during the process of the therapeutic intervention; receiving a cycle of steroids, either intravenously or orally, 6 months prior to the commencement of the assessment protocol and within the study period of intervention; receiving treatment with botulinum toxin in the 6 months prior to the beginning of the study; or the presence of visual disorders noncorrected by optical devices.

This protocol was approved by the local ethics committee of the Rey Juan Carlos University. Informed consent was obtained from all participants included in this study.

### Intervention

All groups received the intervention between November and January of 2019. Both the EG and the CG received two 60 min sessions per week over a ten-week period (a total of 20 sessions for each group).

CG received a specific UL intervention by two physical therapists based on conventional motor rehabilitation therapy (60 min) based on shoulder, elbow, wrist, and finger mobilization, strengthening of UL extensor muscles and stretching exercises for UL flexor muscles [7, 8], and with functional task practice trying to imitate the movements of the serious games designed for the experimental group (i.e. reaching movements, dexterity, grasping and pincer grasp movements using objects of daily living, such as coins, keys, balls, cups, plates) [10, 11].

The EG received the same conventional motor rehabilitation therapy (45 min) plus LMC (15 min) by two physical therapists while seated at a table placed at mid-trunk height and with the elbow placed at an initial 90° elbow flexion. When necessary, manual assistance by the physical therapist was provided.

All interventions took into account the degree of fatigue experienced by the patients introducing rest time periods.

### Development tools

The video games presented in this paper were developed using Unity3D Game Engine software. A small USB peripheral device that supports hand and finger movements as an input denoted as the Leap Motion Controller (LMC) was chosen as the hand motion capture sensor. This sensor offers excellent precision when tracking different parts of the hand, including movements and positions of the joints of the fingers and the palm of the hand. It must be highlighted that the LMC does not require hand contact or touching for interaction.

#### Description of the video games

**Fig. 1:**
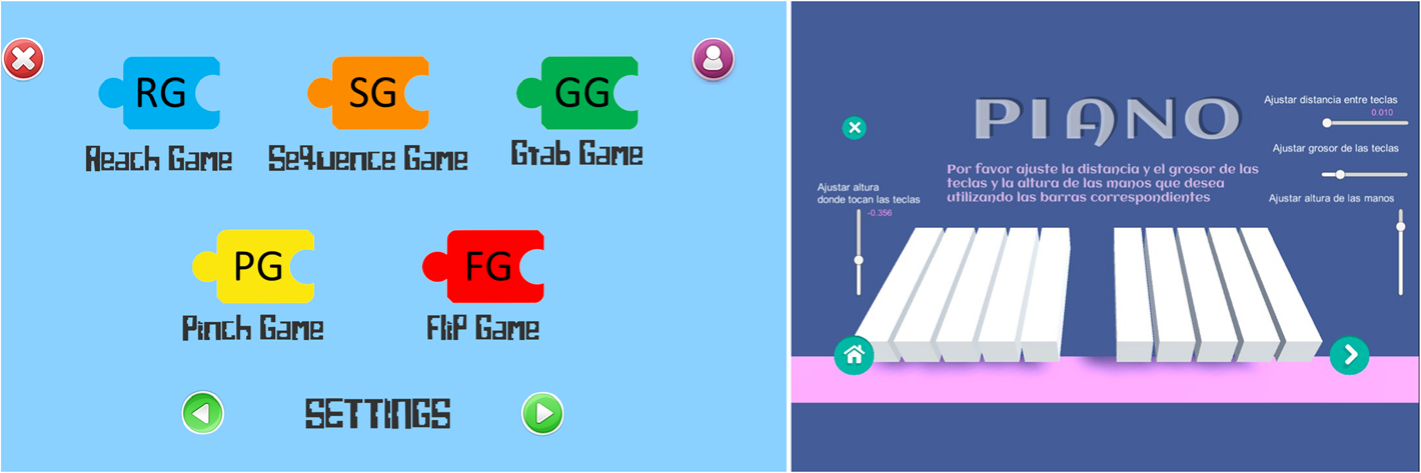
Set of video games designed for the Leap Motion® System used in this protocol. Screen translation: Please adjust the distance and thickness of the keys and the height of the hands as you want, using the corresponding bars. Height, distance and thickness of the keys

**Fig. 2:**
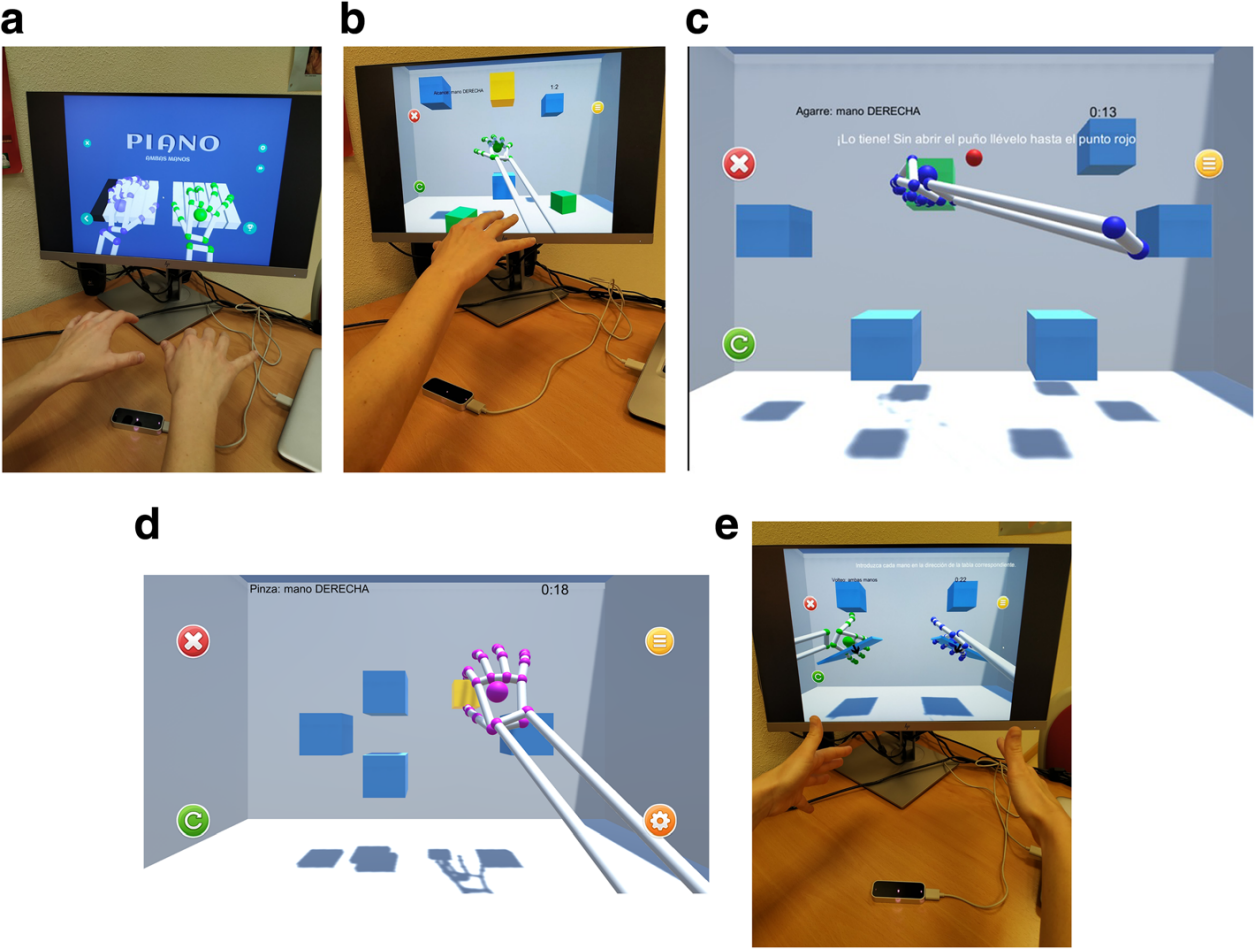
**a** Piano Game. Screen translation: Piano: both hands. **b** Reach Game. Screen translation: Reach: right hand. **c** Grab Game. Screen translation: Grab: right hand. You caught it! without opening your hand, transport the cube to the red point. **d** Pinch Game. Screen translation: Pinch: right hand. **e.** Flip Game. Screen translation: Flip: both hands. Please place your hand in the corresponding blue table position

For this study, six serious games were developed by the UC3M authors, according to the guidelines provided by clinicians. The development of each video game aimed to imitate exercises and movements commonly included in conventional rehabilitation, such as palmar prehension, finger flexion and extension, or hand pronation-supination Additionally, some cognitive load when training was included through memory exercises. The Leap Motion sensor was employed to capture the user’s hand movements, and different virtual environments were created using Unity3D Game Engine software. In total, six video games were developed. The games were performed firstly unilaterally (each hand separately) and then bilaterally (both hands at the same time). Figure 1 presents the whole set of video games used in this protocol: the Piano Game (PI), the Reach Game (RG), the Sequence Game (SG), the Grasp Game (GG), the Pinch Game (PG), and the Flip Game (FG). A full description of these games is provided in a previous study [7]. However, the main features of each game are described as follows:

PI: This serious game represents a virtual piano keyboard with ten keys, each corresponding to a single finger on each hand (see Fig. 2a). The user must play the piano key that is illuminated with the corresponding finger. During the game, the keys light up first in an orderly sequence, from the little finger to the thumb, and then in a random sequence. For each key pressed correctly, one point is added to the total score. Higher scores equate to better game performance.

RG: This serious game encourages the user to reach for several cubes shown in different spatial positions, placed within the reaching range of the user’s upper extremity (see Fig. 2b). A highlighted cube indicates the target to be touched. When the user reaches the cube, it falls to the floor of the virtual scene. The cubes are randomly highlighted after a target is reached. The cubes on the screen are placed at different heights and depths within the user’s workspace. To complete the game, the user must reach all cubes.

SG: This serious game uses the same scenario as the RG game. The user observes a certain sequence of cubes, which is then reproduced through a color change to the cubes that appear on the screen; the user must memorize the sequence and subsequently reach for the cubes in the same order. Furthermore, this game includes exercising visual sequential memory as well as the physical skills that are trained by the RG.

GG: This game encourages the patient to perform movements of closing and opening of the hand (grasping) in coordination with reaching movements. For that purpose, a set of cubes are arranged in a specific pattern, including a red circle in the central part of the screen (see Fig. 2c). When a cube is highlighted, the user must grasp the cube and move it to the red circle while keeping their fist closed. Once the cube and the red circle come into contact, the user must open the hand with all the fingers stretched to release the cube. The cube may only be released when it touches the red circle.

PG: This game was designed to improve bidigital grip through the performance of a pinching movement between the thumb and the index fingers. As in the previous games, a cube highlighted is presented in the center of the screen and the user must make the cube smaller, using a pinching movement, until the cube disappears (see Fig. 2d).

FG: In this video game, the user must place the palm of the hand over the Leap Motion device imitating a waiter holding out a tray (Fig. 2e). A small tray with a cube appears in the center of the screen. The patient must turn the palm downwards. Doing this tray rotation, the cube detaches from tray and it falls to the bottom of the screen.

Altogether, the use of these video games aims to be as non-exclusionary as possible. The games are easy to customize according to patient and rehabilitation needs. For that purpose, a setting menu is included in each videogame in order to set the parameters to best fit the user’s capabilities or limitations. The settings can be defined by therapists at the beginning of the training session, or during the performance of the video game.

#### Measures

All assessments were performed by three physical therapists trained in the use of the measures (each therapist always performed the same measures with all patients in all the evaluation periods) and blinded to the intervention received by the subjects. The following outcome measures were used in both groups, both at the beginning, at the end of the intervention and in a follow-up period of 1 month without receiving any treatment for both groups.

*Grip strength.* A Jamar® hydraulic hand dynamometer was used to measure grip strength. This dynamometer offers accurate and repeatable grip strength readings scaled in pounds and kilograms. All the patients performed three grip movements, and the mean values were recorded. The data for the less and more affected sides were recorded in kilograms. The Jamar® hydraulic hand dynamometer is one of the most commonly used objective tools to assess grip strength, and is considered a device of excellent reliability, sensitivity, and ease of use. It is recommended by the American Society of Hand Therapists and by the Brazilian Society of Hand Therapists [12].

*The Box and Blocks Test (BBT)* was performed to measure unilateral gross manual dexterity on both the less and more affected side. The BBT consists of moving the maximum number of blocks from one compartment of a box to another, one by one, within one minute. The BBT is a quick, simple, and reliable measurement of manual dexterity. Its administration procedure is standardized, and its validity has been shown in elderly subjects with upper limb disability [13, 14].

*The Purdue Pegboard Test (PPT)* was used for the assessment of fine manual dexterity, gross dexterity, and coordination. This test evaluates the speed and motor dexterity of each hand and the manual dexterity using both hands at the same time. The PPT features a board with two columns with 25 holes each and a specific number of pins, washers, and collars placed in four containers across the top of the board. The test consists of inserting as many pins as possible in three distinct phases, with a time limit of 30 s for each part. First, the test is performed with the dominant hand, then with the non-dominant hand, and then with both hands at the same time. The number of pins inserted is recorded [15, 16].

*Nine Hole Peg Test (NHPT)* was used. It is a hand function test, which consists of a plastic peg board (25.0 cm × 12.7 cm × 2.3 cm) with nine holes (2.54 cm between the holes) and nine pegs (3.2 cm long, 0.64 cm wide). The participant has to put the nine pegs in the peg board as fast as possible, one at a time with one hand only, and then remove them again. The test is performed two times per hand, with the non-affected hand first. The time it takes to fulfill the second trial with the more-affected hand is used for the analysis [17].

*Fatigue Severity Scale (FSS).* The FSS described by Krupp et al., [18] is one of the most commonly used scales for the assessment of fatigue in MS attributed to a multifactorial origin. It consists of nine items that are assessed by the patient with a score between 0 and 7. The cut-off point of this scale is arbitrary, with a score of 5 used by most authors as the reference value to distinguish the presence or absence of the symptom. The result is interpreted as a percentage measure.

*Multiple Sclerosis Impact Scale (MSIS-29).* This scale is a specific instrument that allows for assessing the physical and psychological well-being of subjects with MS. It is made up of 29 questions divided into two components: a physical magnitude comprising the first 20 questions, and a psychological magnitude with the last 9 questions. The answers are scored on a Likert scale from 1 to 5, with a maximum of 100 points in the physical part and 45 points in the psychological evaluation [19]. The results are interpreted as a percentage measure. The MSIS-29 has demonstrated its validity and suitability for the evaluation of people with MS, compared to other established measures [20]. It is considered a reliable method that assesses quality of life within the field of MS [19]. The MSIS-29 scale is within the 20 specific scales validated for the measurement of quality of life in the context of MS and is among the three most commonly used according to a number of articles published in this regard [21].

*Satisfaction and adherence.* The Client Satisfaction Questionnaire (CSQ-8) was used to evaluate the satisfaction of health service users for both groups. This is a self-administered post-treatment questionnaire, comprised of eight items that evaluate the level of satisfaction regarding the care and quality of the service received and the level of fulfillment of the patient’s expectations regarding the treatment administered. The total score of the questionnaire is 32 points, with higher values meaning higher satisfaction with the treatment received [22, 23]. The result is calculated as a percentage measure. In addition, the EG completed a satisfaction questionnaire experimental related to the LMC treatment program. It was designed by the research group based on previous publications on using video games in MS [24]. The questionnaire was composed of 18 items that assess the degree of satisfaction in the following dimensions: technical quality and operation of the equipment (4 items); ease of the video game to be played even in disadvantageous conditions (5 items); program compliance and satisfaction in relation to the treatment performed and its applicability (7 items); general degree of satisfaction or complacency (2 items). The answers of this questionnaire are established on a five-point Likert scale, from not satisfied (= 1) to very satisfied (= 5), with alternative directionality to reduce stereotyped responses. Regarding the interpretation of the results of the surveys, the total score was calculated as a percentage measure.

Additionally, we recorded the attendance rate (%) for therapy sessions (compliance).

### Statistical analysis

The statistical analysis was performed using the SPSS statistical software system (SPSS Inc., Chicago, IL; version 22.0). The Shapiro-Wilk test was used to screen all data for normality of distribution. The Friedman test was used, which is a non-parametric test for repeated measurements in related samples. A Bonferroni correction was performed to adjust for multiple testing. With 3 comparisons, a *p* value < 0.016 was considered statistically significant. In the event that there were significant differences, the Wilcoxon test was performed, which allows two related samples to be compared. Additionally, the Mann-Whitney test for non-related samples was used to compare variables, significant values were considered as *p* < 0.05.

## Results

The sample consisted of a total of 30 patients, 12 male and 18 female, of the 32 selected at the study onset. Two subjects were excluded to due to a fall injury and outbreak, respectively. The age of the patients ranged from 26 to 66 years (mean age 46.66 ± 2.04 years). In 17 patients, the more affected side was on the right, whereas the left side was the most affected for the remaining 13 patients. The types of the MS were relapsing-remitting MS (RR-MS) in 11 patients, secondary-progressive MS (SP-MS) in 13 patients and primary-progressive MS (PP-MS) in 6 patients. The evolution time was 13.29 ± 1.68 years. The mean score on the EDSS scale was 5.44 ± 0.23. The patients were randomly assigned into two groups, 16 of whom were assigned to the experimental group while 14 were assigned to the control group (Table 1). There were no statistically significant differences regarding age (*p* = 0.107), disease duration (*p* = 0.281) and EDSS (*p* = 0.903) between control and experimental group. Intergroup and within-group statistical analysis are summarized in Tables 2 and 3.

**Table 1:** Patient features

Groups (n)	Age (years) Mean (±Standard deviation)	Gender	More affected side	Type of MS	Disease duration (years) Mean (±Standard deviation)	EDSS Mean (±Standard deviation)
**Experimental group (16)**	49.86 (±2.46)	7 Male 9 Female	7 Right 9 Left	RRMS: 4 SPMS: 8 PPMS: 4	15.20 (±2.43)	5.43 (±0.31)
**Control group (14)**	42.66 (±3.14)	5 Male 9 Female	6 Right 8 Left	RRMS: 7 SPMS: 5 PPMS: 2	10.91 (±2.19)	5.45 (±0.36)

*EDSS* Kurtzke Expanded Disability Status Scale; *MS* Multiple Sclerosis; *PP-MS* Primary-Progressive MS; *RR-MS* Relapsing-Remitting MS; *SP-MS* Secondary-Progressive MS. Data are expressed as mean (±Standard deviation)

**Table 2:** Comparison of outcome scores (Intragroup analysis)

Variable		Pre	Post	Follow up	Intragroup analysis *p*-value	Two-paired comparisons		
		Median (Interquartile range)						
						Pre vs. Post *p*-value	Pre vs. Follow up *p*-value	Post vs. Follow up *p*-value
Jamar More affected	Experimental group	17.00 (20.34)	21.00 (13.33)	17.33 (12.00)	.038	.099	.670	.092
	Control group	13.00 (11.00)	13.00 (10.33)	13.00 (7.67)	.208	.070	.448	.091
Jamar Less affected	Experimental group	21.33 (16.00)	20.00 (19.67)	18.33 (16.00)	.011*	.019†	.776	.002†
	Control group	17.00 (11.33)	16.33 (14.50)	15.33 (14.00)	.337	.723	.123	.063
PPT More affected	Experimental group	7.66 (4.33)	9.66 (5.33)	9.00 (4.66)	.015*	.008†	.157	.055
	Control group	3.33 (8.83)	3.66 (7.67)	3.66 (10.50)	.227	.514	.204	.203
PPT Less affected	Experimental group	9.33 (3.33)	10.33 (3.00)	9.66 (3.34)	.006*	.004†	.220	.110
	Control group	9.66 (6.33)	10.00 (11.00)	10.00 (11.00)	.191	.384	.375	.227
PPT Both hands	Experimental group	12.66 (5.33)	15.33 (8.00)	14.66 (6.00)	.014*	.005†	.441	.480
	Control group	9.33 (12.66)	6.66 (13.67)	7.33 (12.66)	.966	.339	.932	.496
PPT Assembly	Experimental group	15.66 (5.00)	20.00 (7.33)	16.66 (7.66)	.002*	.001†	.038†	.094
	Control group	13.33 (18.83)	10.66 (15.83)	14.33 (19.17)	.104	.440	.128	.080
BBT More affected	Experimental group	40.00 (23.00)	48.00 (14.00)	51.00 (9.00)	.002*	.003†	.016†	.779
	Control group	26.00 (31.50)	28.00 (30.00)	30.00 (25.00)	.140	.071	.090	.779
BBT Less affected	Experimental group	44.00 (18.00)	48.00 (13.00)	52.00 (12.00)	.002*	.028†	.002†	.107
	Control group	41.00 (27.00)	42.00 (36.00)	48.00 (36.00)	.008*	.007†	.013†	.236
NHPT More affected	Experimental group	35.35 (13.09)	32.03 (19.74)	28.90 (15.21)	.127	.334	.088	.211
	Control group	60.22 (57.33)	70.15 (76.02)	70.15 (70.19)	.122	.929	.182	.236
NHPT Less affected	Experimental group	30.62 (16.95)	29.07 (15.23)	28.15 (11.10)	.282	.078	.053	.820
	Control group	27.50 (24.06)	23.31 (19.27)	25.24 (18.50)	.053	.138	.172	.236
MSIS-29 physical score	Experimental group	62.00 (36.00)	60.00 (22.00)	63.00 (34.00)	.701	.233	.629	.826
	Control group	55.00 (33.00)	66.00 (31.00)	66.00 (34.00)	.917	.527	.789	.372
MSIS-29 psychological score	Experimental group	57.77 (28.89)	64.44 (20.00)	66.66 (35.56)	.878	.552	.780	.683
	Control group	46.66 (23.33)	51.11 (16.67)	48.00 (28.28)	.186	.086	.723	.210
FFS	Experimental group	58.73 (37.88)	63.49 (42.86)	58.73 (17.46)	.819	.363	.629	.712
	Control group	57.14 (26.19)	58.73 (17.47)	58.73 (30.17)	.679	.700	.752	.959

*PPT* Purdue Pegboard Test; *BBT* box and block test; *NHPT* Nine Hole Peg Test. *MSIS-29* Multiple Sclerosis Impact Scale. *FSS* Fatigue Severity Scale. Data are expressed as median and interquartile range. *p value < 0.016 using the Friedman test (after Bonferroni correction) and †*p* value < 0.05 using the Wilcoxon test for related samples

**Table 3:** Comparison of outcome scores between the experimental and control group (Intergroup analysis)

Variable	Experimental group Median (Interquartile range)			Control group Median (Interquartile range)			Experimental vs. Control group		
	Pre	Post	Follow up	Pre	Post	Follow up	Pre *p*-value	Post *p*-value	Follow up *p*-value
Jamar More affected	17.00 (20.34)	21.00 (13.33)	17.33 (12.00)	13.00 (11.00)	13.00 (10.33)	13.00 (7.67)	0.259	0.092	0.097
Jamar Less affected	21.33 (16.00)	20.00 (19.67)	18.33 (16.00)	17.00 (11.33)	16.33 (14.50)	15.33 (14.00)	0.982	0.181	0.278
PPT More affected	7.66 (4.33)	9.66 (5.33)	9.00 (4.66)	3.33 (8.83)	3.66 (7.67)	3.66 (10.50)	0.133	0.032*	0.221
PPT Less affected	9.33 (3.33)	10.33 (3.00)	9.66 (3.34)	9.66 (6.33)	10.00 (11.00)	10.00 (11.00)	0.729	0.277	0.746
PPT Both hands	12.66 (5.33)	15.33 (8.00)	14.66 (6.00)	9.33 (12.66)	6.66 (13.67)	7.33 (12.66)	0.071	0.019*	0.067
PPT Assembly	15.66 (5.00)	20.00 (7.33)	16.66 (7.66)	13.33 (18.83)	10.66 (15.83)	14.33 (19.17)	0.332	0.008*	0.460
BBT More affected	40.00 (23.00)	48.00 (14.00)	51.00 (9.00)	26.00 (31.50)	28.00 (30.00)	30.00 (25.00)	0.068	0.036*	0.010*
BBT Less affected	44.00 (18.00)	48.00 (13.00)	52.00 (12.00)	41.00 (27.00)	42.00 (36.00)	48.00 (36.00)	0.356	0.549	0.321
NHPT More affected	35.35 (13.09)	32.03 (19.74)	28.90 (15.21)	60.22 (57.33)	70.15 (76.02)	70.15 (70.19)	0.122	0.069	0.011*
NHPT Less affected	30.62 (16.95)	29.07 (15.23)	28.15 (11.10)	27.50 (24.06)	23.31 (19.27)	25.24 (18.50)	0.278	0.160	0.205
MSIS-29 physical score	62.00 (36.00)	60.00 (22.00)	63.00 (34.00)	55.00 (33.00)	66.00 (31.00)	66.00 (34.00)	0.381	0.475	0.628
MSIS-29 psychological score	57.77 (28.89)	64.44 (20.00)	66.66 (35.56)	46.66 (23.33)	51.11 (16.67)	48.00 (28.28)	0.075	0.127	0.174
FSS	58.73 (37.88)	63.49 (42.86)	58.73 (17.46)	57.14 (26.19)	58.73 (17.47)	58.73 (30.17)	0.729	0.644	0.963

*PPT* Purdue Pegboard Test; *BBT* box and block test; *NHPT* Nine Hole Peg Test. *MSIS-29* Multiple Sclerosis Impact Scale. *FSS* Fatigue Severity Scale. Data are expressed as median and interquartile range. *p value < 0.05 using Mann-Whitney test for not related sample

The within-group statistical analysis for the experimental group showed significant improvements in all post-treatment compared to pre-treatment assessments, except for the Jamar score on the more affected side, the NHPT on both sides, the MSIS 29 for both scores, and the FSS. Significant improvements were observed on the Jamar for the less affected side (*p* = .019); the PPT for the more affected side (*p* = .008), the PPT for the less affected side (*p* = .004), the PPT both hands (*p* = .005) and the PPT assembly (*p* = .001); the BBT for the more affected side (*p* = .003), and the BBT for the less affected side (*p* = .028). These results mean that patients improved their scores in post-treatment measurements. Furthermore, for the follow-up measures compared to the pre-treatment assessments, significant improvements were found for the PPT assemblies (*p* = .038), the BBT for the more affected side (*p* = .016), and the BBT for the less affected side (*p* = .002). These results show that patients improved their follow-up scores, compared to pre-treatment measurements. Also, for the follow-up compared to the post-treatment measurements, statistically significant differences were found in the Jamar score for the less affected side (*p* = .002). These results suggest that patients scores decreased during follow-up, compared to the post-treatment scores (Table 2).

In the intra-group statistical analysis for the control group, significant improvements were only observed for the BBT on the less affected side (*p* = .007) in the post-treatment compared to pre-treatment assessments and the BBT on the less affected side (*p* = .013) in the follow-up compared to pre-treatment assessments (Table 2).

According to the statistical inter-group analysis, no significant differences were observed between either of the two groups in terms of baseline clinical characteristics. In the post- treatment evaluation, significant improvements were found for the PPT on the more affected side (*p* = .032), the PPT both hands (*p* = .019), the PPT assembly (*p* = .008), and the BBT on the more affected side (*p* = .036). These results mean that patients in the experimental group improved their scores in post-treatment measurements, compared to the control group. In addition, for the follow-up measurements, significant improvements were found for the BBT on the more affected side (*p* = .010) and the NHPT on the more affected side (*p* = .011). These outcomes show that patients in the experimental group improved their scores in follow-up measures, when compared to the control group (Table 3).

Furthermore, compliance to the interventions was excellent (100%) and no adverse side-effects were observed for both groups.

All patients, those in the control group (89.18 ± 2.49) and those in the experimental group (89.91 ± 2.13), showed high scores for satisfaction, measured using the CSQ8 scale. Regarding the scale of satisfaction with the technology, the experimental group obtained an average of 81.45 ± 2.52, indicating that the patients were very satisfied with the virtual treatment.

## Discussion

The purpose of this study was to evaluate the effectiveness of the LMC based Serious Games specifically designed for the UL in people with MS. In the experimental group compared to the control group, significant improvements were observed in the post-treatment assessment for the PPT on the more affected side, PPT for both hands, PPT assembly, and BBT on the more affected side. Also, significant results were found in the follow-up in the BBT and NHPT for the more affected side, although these improvements should be interpreted with caution because the two groups started from different clinical performance. Furthermore, intra-group analysis for the EG showed significant improvements for all measures except for NHPT, MSIS-29, and FSS.

Few studies have been conducted for UL rehabilitation in MS patients. Jonsdottir et al. [3] studied the feasibility and efficacy of a serious games approach to supervised UL rehabilitation in 18 MS patients, showing improvements in dexterity assessed with the BBT and NHPT with a positive impact on the mental domain of perceived health. Jonsdottir et al. [25] also studied the feasibility of serious games platform using Kinect compared to exergames using the Nintendo Wii for UL in 16 people with MS. Their results showed clinically important improvements in fine and gross hand function evaluated by BBT and NHPT after the experimental intervention with serious games with Kinect. Waliño-Paniagua et al. [6] assessed occupational therapy plus VR via a webcam for UL rehabilitation in 16 MS patients and showed a tendency towards statistical significance related to motor dexterity measured by PPT, the Jebsen-Taylor Hand Function Test and the Grooved Pegboard Test.

To our knowledge, this is the first RCT to evaluate grip muscle strength, coordination, speed of movements, fine and gross dexterity, fatigue, and quality of life after using serious games designed for neurological diseases with the LMC system for UL rehabilitation MS patients. Webster et al. [26] assessed the opinion of five MS patients using focus groups regarding the development of virtual environments using LMC. The authors compiled information regarding UL dysfunctions, participant experiences, and their comfort with hand tracking technology. The differing opinions regarding preferable exercises also demonstrated the need for choice and personalization in rehabilitation game design for UL using LMC. However, this qualitative study did not use objective UL measures to assess MS patients after the treatment and follow-up period.

Our experimental protocol was based in two 60-min sessions per week over a ten-week period of conventional motor rehabilitation therapy (45 min) plus LMC (15 min) with a total of 20 sessions. There are no other studies available to compare our treatment protocol based on LMC. However, Jonsdottir et al. [3] used 12 sessions, 3–5 times per week, lasting 45 min using the Kinect combined with conventional rehabilitation. Jonsdottir et al. [25] used 12 sessions, 40 min per session, 4–5 sessions per week, using a Kinect or Nintendo Wii console combined with conventional rehabilitation. Finally, Waliño-Paniagua et al. [6] used 20 sessions of occupational therapy, lasting 30 min, twice weekly, plus 20 min of VR via a webcam. These results suggest that at least 12 sessions with a virtual environment combined with conventional rehabilitation are necessary to achieve dexterity improvements in MS patients. However, specific serious games are necessary for the UL rehabilitation in patients with MS, since Waliño-Paniagua et al. [6] did not achieve significant improvements with their protocol.

Our results showed improvements in the post-treatment assessment for unilateral gross manual dexterity, fine manual dexterity, and coordination. These findings seemed to be more outstanding on the more affected side. Our results are in line with other studies that have employed LMC in neurological diseases [7, 27–32]. However, our results did not show improvements in physical and psychological well-being and fatigue perceived by the MS patients. These results may be due to the duration of the experimental protocol designed, as well as the variable nature of the MS and the multudimensionality of the fatigue and quality of life constructs.

Furthermore, satisfaction with the experimental treatment showed high scores measured using the CSQ8 scale. In addition, the scale of satisfaction with the technology designed by the research team showed excellent results. Lack of motivation is a common problem in long-term rehabilitation, leading to reduced adherence. Training in VR can provide tailored environments and the opportunity to solve motor problems in a gaming environment, with the potential of enhancing motivation and to perform repetitive tasks. The motivation factor and the fact that active gaming elicits more arm movement repetitions than traditional rehabilitation [33] could justify the using of this type of intervention combined with conventional rehabilitation approaches due to the excellent satisfaction results achieved. Also, our findings could highlight specific opportunities for LMC system and the serious games designed for UL rehabilitation in MS patients with EDSS scores of 3.5–7.5. So, future studies should be conducted as an at-home rehabilitation system to corroborate this potential improvements.

This study presents several limitations. First, the results cannot be generalized for all patients with MS, so it is necessary to interpret these findings with caution regarding people with MS with different EDSS scores, type of MS and disease duration. Moreover, the sampling methods could have resulted in selection bias as patients were recruited from different MS associations. Improvements reached in the follow-up in BBT and NHPT for the more affected side should be interpreted with caution because the two groups started from different clinical performance. Additionally, further RCT comparing our experimental protocol with other conventional approaches for UL rehabilitation are required to verify these results.

## Conclusions

An experimental protocol using an LMC system and serious games designed for UL rehabilitation plus conventional motor rehabilitation therapy showed improvements for unilateral gross manual dexterity, fine manual dexterity, and coordination in MS patients with EDSS scores of 3.5–7.5 with high satisfaction and excellent compliance. These findings were more outstanding in the more affected side. Future studies are necessary to corroborate our findings.

## Data Availability

All the data and materials could be found at Faculty of Health Sciences of Rey Juan Carlos University.

## Abbreviations

ADL: Activities of daily living
BBT: Box and Blocks Test
CSQ-8: Client Satisfaction Questionnaire
CG: Control group
EG: Experimental group
FSS: Fatigue Severity Scale
FG: Flip Game
GG: Grasp Game
EDSS: Kurtzke Expanded Disability Status Scale
LMC: Leap Motion Controller
MS: Multiple sclerosis
MSIS-29: Multiple Sclerosis Impact Scale
NHPT: Nine Hole Peg Test
PI: Piano Game
PG: Pinch Game
PP-MS: Primary-Progressive MS
PPT: Purdue Pegboard Test
RCT: Radomized Controlled Trial
RG: Reach Game
RR-MS: Relapsing-Remitting MS
SP-MS: Secondary-Progressive MS
SG: Sequence Game
UL: Upper limb
VR: Virtual reality
